# Emerging Therapeutic Targets for Portal Hypertension

**DOI:** 10.1007/s11901-023-00598-4

**Published:** 2023-02-11

**Authors:** Eric Felli, Yelidousi Nulan, Sonia Selicean, Cong Wang, Jordi Gracia-Sancho, Jaume Bosch

**Affiliations:** 1 Department of Visceral Surgery and Medicine, Inselspital, Bern University Hospital, University of Bern, 3012 Bern, Switzerland; 2 Department for BioMedical Research, Hepatology, University of Bern, 3012 Bern, Switzerland; 3 Liver Vascular Biology Research Group, CIBEREHD, IDIBAPS Research Institute, 08036 Barcelona, Spain

**Keywords:** Portal hypertension, Cirrhosis of the liver, Fibrosis, Endothelial dysfunction, Hepatic vascular resistance

## Abstract

**Purpose of Review:**

Portal hypertension is responsible of the main complications of cirrhosis, which carries a high mortality. Recent treatments have improved prognosis, but this is still far from ideal. This paper reviews new potential therapeutic targets unveiled by advances of key pathophysiologic processes.

**Recent Findings:**

Recent research highlighted the importance of suppressing etiologic factors and a safe lifestyle and outlined new mechanisms modulating portal pressure. These include intrahepatic abnormalities linked to inflammation, fibrogenesis, vascular occlusion, parenchymal extinction, and angiogenesis; impaired regeneration; increased hepatic vascular tone due to sinusoidal endothelial dysfunction with insufficient NO availability; and paracrine liver cell crosstalk. Moreover, pathways such as the gut-liver axis modulate splanchnic vasodilatation and systemic inflammation, exacerbate liver fibrosis, and are being targeted by therapy. We have summarized studies of new agents addressing these targets.

**Summary:**

New agents, alone or in combination, allow acting in complementary mechanisms offering a more profound effect on portal hypertension while simultaneously limiting disease progression and favoring regression of fibrosis and of cirrhosis. Major changes in treatment paradigms are anticipated.

## Introduction

Portal hypertension (PH) is a major consequence of liver cirrhosis (LC) where its complications are key determining prognosis [1]. These complications include the development of esophageal varices (that can rupture causing massive gastrointestinal bleeding), formation of ascites and its complications (spontaneous bacterial peritonitis, hepatorenal syndrome), and hepatic encephalopathy. The prognosis of cirrhosis is good before the development of complications related to PH. In this stage of compensated cirrhosis, the median survival is about 12 years. However, cirrhosis becomes a highly lethal disease, with a median survival of about 2 years once the patients have developed ascites, bleeding, or encephalopathy, which is known as the decompensated stage of the disease [2]. The two main mechanisms of PH are increased intrahepatic vascular resistance (IHVR) and splanchnic vasodilatation with increased inflow of blood in the portal system [3]. IHVR (produced by structural remodeling of the liver architecture, increasing hepatic vascular tone due to hepatic endothelial dysfunction) is the initiating and more important factor determining PH, while splanchnic hyperemia develops later, as an adaptive response to maintain liver perfusion challenged by the development of portal-systemic collaterals and by the impaired metabolic exchange at the disturbed hepatic microcirculation. In advanced stages, the splanchnic vasodilation represents an important mechanism maintaining and aggravating PH. Moreover, as splanchnic vasodilation progresses, it determines a state of “effective hypovolemia” that leads to systemic hypotension, expansion of the plasma volume and increased cardiac output, the so-called hyperdynamic syndrome of cirrhosis, which plays an important role in the pathophysiology of ascites and associated renal abnormalities (hyponatremia, hepatorenal syndrome, acute kidney injury). Of note, in decompensated cirrhosis, systemic inflammation becomes an important factor driving progression of cirrhosis to further decompensation, acute-on-chronic liver failure (ACLF) and death or liver transplantation.

PH becomes clinically significant (CSPH) when the porto-caval pressure gradient, measured clinically by the hepatic vein pressure gradient (HVPG) is equal or above 10 mmHg [4]. Patients with CSPH are at increased risk of developing decompensation [5] and hepatocellular carcinoma [6, 7]. The importance of subclinical PH (HVPG from 5.5 to 9.5 mmHg) is still a matter of debate [8] but nonetheless indicates underlying severe liver disease.

Cirrhosis of the liver is responsible of about 90% of cases of PH in western countries, so we will focus on this disease. Cirrhosis is the result of a prolonged and progressive scarring process (liver fibrosis), caused by chronic injury and inflammation. Fibrosis leads to marked architectural liver disturbances, which are aggravated by loss of parenchymal cells due to vascular occlusion and ensuing tissue collapse, which makes that attempts of regeneration lead to the formation of regenerating nodules separated by fibrous tracts, which represents the hallmark of cirrhosis. Impaired liver regeneration and continued fibrogenesis further aggravate the process. Recent evidence suggests that cirrhosis is potentially reversible, but is not yet defined if, why, and when cirrhosis reaches a point of no-return [9, 10]. Reversibility is possible before CSPH, but unlikely after decompensation or in the presence of co-morbidities.

Treatment of PH at early stages is based primarily on controlling the etiological cause of the liver disease, to enhance the chance of reversing the architectural disturbances leading to PH. In addition, several studies have highlighted the role of relatively simple measures such as a safe lifestyle [11] (including maintaining a body mass index between 18 and 28, abstaining from alcohol, and moderate aerobic exercise), which favorably influence liver disease and PH. In addition, agents with the potential to enhance fibrosis regression may be paramount to achieve clinical improvement.

Current pharmacological treatment for PH is based on the use of drugs that act mainly on the increased portal vein inflow, which for short treatments include the intravenous drugs terlipressin [12] (a long-acting vasopressin analog), somatostatin [13], and analogs (octreotide) [14], while for long-term administration, the key agents are still the non-selective beta-blockers propranolol [15], nadolol [16], and, more recently, carvedilol [17, 18]. Despite the marked impact of these drugs ameliorating the prognosis of PH, still a substantial fraction of patients are insufficiently covered by them and require the association of other drugs or of endoscopic, interventional radiology, or surgical procedures.

It is remarkable that to date, not a single agent acting on IHVR has been approved for PH. Novel therapeutic approaches cover a wide range of targets in rapid development. This review focuses on the new agents under development or being studied for repurposing for PH. Notably, this is specially challenging in the aging population since aging favors disease progression and hampers response to many drugs [19].

## Pathophysiological Basis of Therapy: Intra and Extra-hepatic Targets (Fig. 1)

**Fig. 1:**
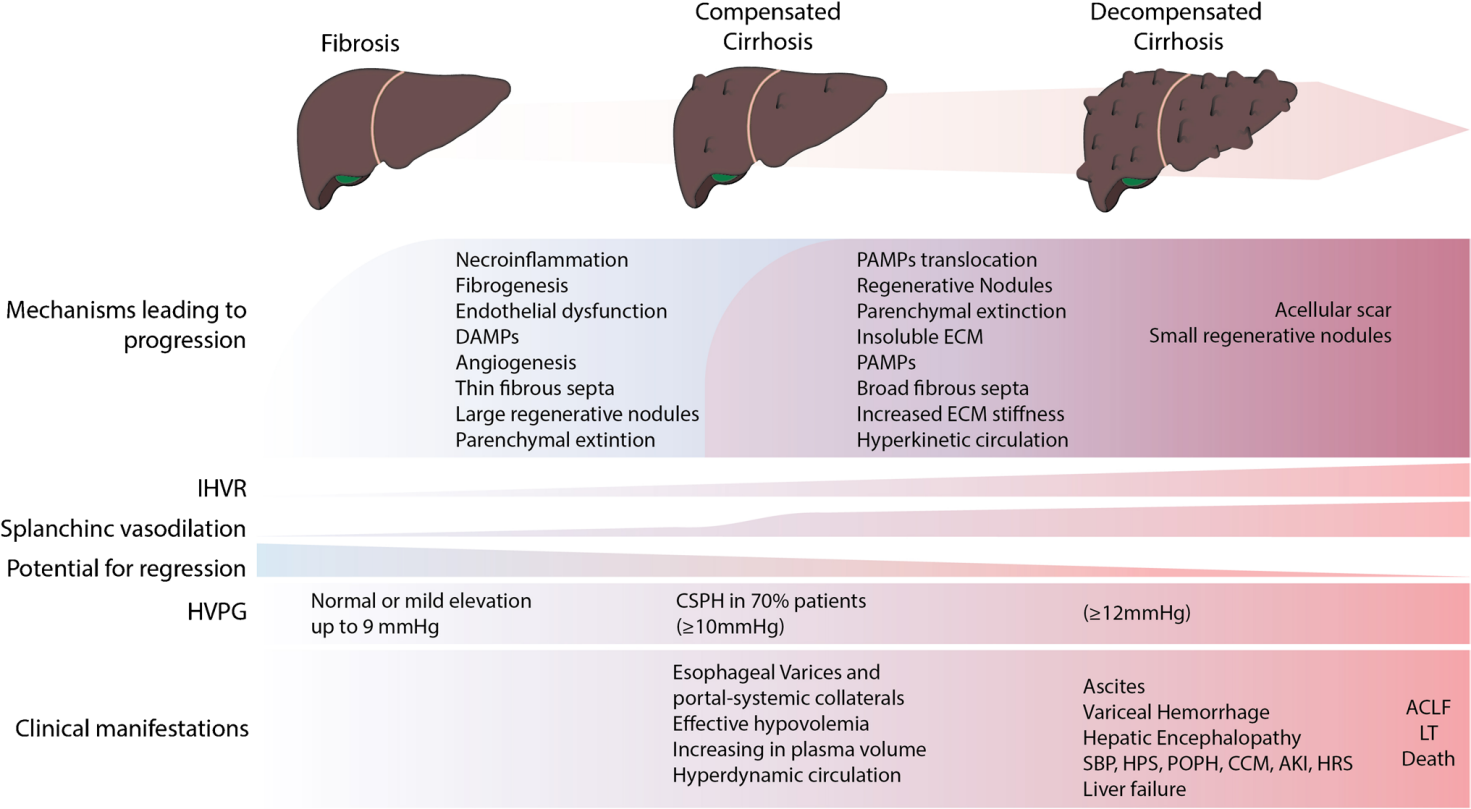
Progression of PH. Liver fibrosis leads progression first to a low-risk, long-lasting stage known as compensated cirrhosis and thereafter to the high-risk and short duration decompensated stage. A series of factors contribute to disease progression and to increased portal pressure. Initially this is mostly linked to fibrogenesis, structural liver changes and increased hepatic vascular tone, while after development of clinically significant portal hypertension (CSPH, defined by a HVPG ≥ 10 mmHg), portal-systemic collaterals, and hyperkinetic circulation, systemic inflammation (prompted among others by abnormalities of the gut-liver axis and translocation of bacterial products, DAMPs and PAMPs) becomes the main factor determining clinical complications and prognosis. Clinical stages also determine the aim of therapy, the choice of therapeutic agents, and the likelihood of regression upon removal or effective treatment of etiological factors. Abbreviations: intrahepatic vascular resistance (IHVR), liver transplantation (LT), acute on chronic liver failure (ACLF), damage associated molecular patterns (DAMPs), pattern associated molecular patterns (PAMPs), extra cellular matrix (ECM), spontaneous bacterial peritonitis (SBP), hepatopulmonary syndrome (HPS), portopulmonary hypertension (POPH), cirrhotic cardiomyopathy (CCM), acute kidney injury (AKI), hepatorenal syndrome (HRS)

As commented, the initial mechanism leading to PH is the IHVR to portal blood flow. This is due to a combination of structural changes and increased vascular tone. The structural component is determined by progressive fibrosis, vascular occlusion with ensuing parenchymal extinction and tissue collapse, which are mediated by chronic liver injury/repair, oxidative stress, release of damage associated molecular patterns (DAMPs) and pro-inflammatory cytokines, activation of hepatic stellate cells (HSCs), and macrophages. These processes can be targeted with specific drugs, which is a relevant area of research.

Splanchnic vasodilation and increased portal inflow represent the main extra-hepatic factor aggravating PH. This is mediated by increased NO availability [20], decreased response to vasoconstrictors [21], and the vasculogenesis in splanchnic organs via VEGF-VEGF-R2 signaling [22]. Splanchnic vasodilation is minimal or absent until development of CSPH. Then, portal-systemic collaterals begin to form, driven by increased portal pressure and VEGF-dependent angiogenesis in areas of anatomical connections between the portal and systemic circulations. Functional and anatomic intrahepatic shunts promote secretion of VEGF, which contributes to the formation of collaterals and to splanchnic vasodilation by stimulating the release of NO at the splanchnic arterioles and promoting splanchnic vasculogenesis [23]. Additionally, translocation of intestinal bacterial products and bacteria to the portal circulation may further contribute to upregulate eNOS and worsen splanchnic vasodilation [24] while enhancing the hepatic inflammation through pathogen-associated molecular patterns (PAMPs) [25]. Other vasodilators, including carbon monoxide [26], glucagon [27], and endocannabinoids [28], may aggravate splanchnic arterial vasodilation [29]. Importantly, the splanchnic vasodilation is not a primary factor in PH, but an adaptive response. Curing liver disease or transplantation results in deactivation of the splanchnic vasodilation.

In addition to structural abnormalities (fibrosis and disruption of liver vascular architecture), an increased liver vascular tone further increases IHVR and is thought to represent about 30–35% of the total IHVR. So, interfering with this component has the potential to decrease portal pressure by over 30%, which should be enough for preventing most complications from PH [3]. The hepatic sinusoid is a very complex network which is highly orchestrated by the liver sinusoidal endothelial cells (LSECs) due to their role as a vasoactive and permeabilized plate between the sinusoidal lumen and the parenchyma [30]. Increased liver vascular tone is mainly caused by LSECs dysfunction [31], which is characterized by decreased release of vasodilatory mediators such as nitric oxide (NO) [32], enhanced release, and response to endogenous vasoconstrictors (including the adrenergic system, renin-angiotensin system, prostanoids, cysteinyl-leukotrienes, thromboxane, and endothelins). Loss of LSECs fenestrations (a process known as pseudo-capillarization) marks an important step in the disease as it is responsible for the impaired oxygenation of hepatocytes, favored by accumulation of ECM components such as fibronectins, laminin, forming a basal membrane (absent in the normal liver sinusoids) that further impair the metabolic exchange between circulating blood and hepatocytes [33]. Oxidative stress, increased levels of asymmetrical dimethylarginine (ADMA) and decreased synthesis of tetrahydrobiopterin further decrease NO availability by uncoupling or inactivating endothelial NO-synthase (eNOS) and NO scavenging. Interfering with the chain of events leading to impair NO availability is therefore a relevant therapeutic target. Besides, dysfunctional LSECs become proliferative, pro-inflammatory, and pro-thrombotic and may contribute to vascular occlusion and parenchymal extinction, which has led to introducing anti-coagulants as potential treatments for PH.

The mechanisms leading to endothelial dysfunction are interconnected with those causing HSCs activation and fibrogenesis due to crosstalk between LSECs and HSCs, thus explaining how drugs improving endothelial dysfunction may also improve fibrogenesis (as it happens with statins and antioxidants). Moreover, some extrahepatic stimuli, specifically from the gut-liver axis, are also involved in activation of HSCs and explain the beneficial effects of Farnesoid X Receptor agonists, such as obeticholic acid. Hepatocytes finally undergo necroptosis; cell death stimulates the release of pro-inflammatory elements and contributes to HSC activation [10]. The vascular architecture is remodeled under mechanical and biochemical stimuli that increase liver stiffness, which may further enhance and perpetuate liver fibrosis in a winch-like process.

Under these driving forces, the liver undergoes dramatic changes, the percentage of fibrotic tissue increasing at the expense of the functional parenchymal mass. Disrupted microcirculation generates areas of the parenchyma that become less perfused and together with parenchymal extinction progressively worsens IHVR. As stated before, initial progression of PH is dominated by structural elements, while thereafter extrahepatic factors increasingly contribute to aggravate PH by increasing both the portal blood inflow and the hepatic vascular tone [34].

## Therapeutic Agents for Liver Cirrhosis and Portal Hypertension

The complications of PH do not appear until the HVPG reaches values ≥ 10 mmHg [35]. During the compensated stage, the aim of the treatment of PH is to prevent decompensation. Protection is maximal when the HVPG is decreased below 10 mmHg, but significant protection is already obtained when HVPG decreases by over 10% of baseline [17]. After decompensation, a greater portal pressure reduction is required to protect from repeat or new complications from PH; protection is significant when achieving an HVPG decrease over 20% of baseline and almost optimal when HVPG decreases ≤ 12 mmHg. Baveno VII recommends a different approach for each sub-stage, starting in compensated cirrhosis without CSPH, followed by compensated patients with CSPH, decompensated patients without ascites, and decompensated patients with ascites.

Current drugs used for clinical management of LC patients are based on general therapy (lifestyle intervention), control of etiological factors, and use of drugs that counteract the splanchnic and systemic vasodilation. Up to now, this is achieved mainly by the oral administration of non-selective beta-blockers (NSBB) (like propranolol, and nadolol) for long-term therapy or somatostatin and analogs (mainly octreotide), and vasopressin derivatives such as terlipressin for short-term administration (for instance, for acute variceal bleeding and hepatorenal syndrome). The most recent therapeutic innovation is carvedilol, a NSBB that has intrinsic anti-adrenergic activity, this makes that it decreases portal venous inflow, but also the intrahepatic vascular tone, resulting in an enhanced reduction in HVPG even at low doses. This makes carvedilol the NSBB of choice [17, 18, 36]. Carvedilol can prevent ascites and clinical decompensation in patients with cirrhosis and PH and also markedly improves survival in meta-analysis. This has represented a change of paradigm in the treatment of PH, which is no longer limited to correct or prevent variceal hemorrhage, but to prevent all the complications of PH. The best use of these agents and current therapeutic strategies for PH management are established by the International Baveno VII Consensus conference [37], the European Association for the Study of the Liver (EASL) guideline for decompensated cirrhosis [38], and the American Association for the Study of Liver Disease (AASLD) [4, 39].

### Intrahepatic Targets

In this section, we discuss promising strategies to reduce structural and functional alterations leading to IHVR; a detailed list of drugs that act on these processes is shown in Table 1.

**Table 1:** Intrahepatic therapeutic compounds

Name	Mechanism	Exp phase	Ref
Sapropterin	Analog of BH4, a cofactor of NO synthase	Phase II	[40]
Udenafil	PDE-5-inhibitor	Phase II	[41]
Ambrisentan	ET-A receptor antagonist	Phase II	[42]
Bq 123	ET-A receptor antagonist	Phase II	[43]
Ifetroban	TXA2 prostanoid receptor antagonist	Phase II	[44]
Emricasan	Caspase Inhibitor, anti-inflammatory, anti-apoptotic	Phase II	[45]
Dark chocolate	Antioxidant, NO release	Phase II	[46]
Resveratrol	Antioxidant, NO release, PPAR gamma	Pre-clinical	[47]
Semaglutide	GLP-1 receptor agonist	Phase II	[48]
Ncx-1000	NO donor	Phase IIa	[49]
Simtuzumab	Monoclonal antibody against LOX2	Phase IIb	[50]
Simvastatin	HMG-CoA reductase inhibitor	Phase III	[51]
Darusentan (LU-135252)	ET-A receptor antagonist	Phase III	[52]
Selonsertib	ASK1 inhibitor	Phase III	[53]
Rivaroxaban	Anticoagulant, FXa inhibitor	Phase III	[54]
Cenicriviroc	Chemokine 2 and 5 receptor antagonist	Phase III	[55]
Macitentan	ET receptor antagonist	Phase IV	[56]
Losartan	Ang II receptor antagonist	Phase IV	[57]
Ascorbic acid	Anti-inflammatory	Phase IV	[58]
Sildenafil	PDE-5-inhibitor	Pilot study	[59]
Vardenafil	PDE-5-inhibitor	Pilot study	[60]
Obeticholic acid	FXR agonist	Pre-clinical	[61]
Cilofexor (Formerly GS-9674)	FXR agonist	Pre-clinical	[62]
Px20606	FXR agonist	Pre-clinical	[63]
Fluvastatin	HMG-CoA inhibitor	Pre-clinical	[64]
Ave 9488	NOS transcription enhancer	Pre-clinical	[65]
Nanoparticles-NO	NO nanodelivery	Pre-clinical	[66]
Riociguat	Increase activity of sGC	Pre-clinical	[67]
Tadalafil	PDE-5-inhibitor	Pre-clinical	[67]
Palosuran	Urotensin II receptor antagonist	Pre-clinical	[68]
Abt-627(A-147627)	ET-A receptor antagonist	Pre-clinical	[69]
Bosentan	ET-1 receptor antagonist	Pre-clinical	[70]
Sb209670	ET-A/B receptor antagonist	Pre-clinical	[71]
A-192621	ET-B receptor antagonist	Pre-clinical	[72]
A‐182086	ET-A/B receptor antagonist	Pre-clinical	[73]
Bq‐788	ET-A/B receptor antagonist	Pre-clinical	[74]
Aspirin	Anti-inflammatory	Pre-clinical	[75]
Celecoxib	COX-2 inhibitor	Pre-clinical	[76]
Nitroflurbiprofen	COX inhibitor with NO-donating properties	Pre-clinical	[77]
Terutroban	Prostaglandin-endoperoxide (TP) receptor blocker	Pre-clinical	[78]
Montelukast	Cys-LT_1_ receptor inhibitor	Pre-clinical	[79]
Candesartan	Ang II-receptor blocker	Pre-clinical	[80]
Sodium ferulate	RhoA/Rho-kinase pathway inhibitor	Pre-clinical	[81]
Tempol	Antioxidant	Pre-clinical	[82]
RMnSOD	Antioxidant	Pre-clinical	[83]
Apocynin	Antioxidants, lipid peroxidation inhibitor	Pre-clinical	[84]
Mitoquinone	Antioxidant	Pre-clinical	[85]
MasR antagonist	Block vasodilatory receptors of the alternate RAS and Mas	Pre-clinical	[86]
Ave0991	Non-peptidic Ang-(1–7) agonist	Pre-clinical	[87]
Ag490	JAK2 inhibitor	Pre-clinical	[88]
Spironolactone	Aldosterone receptor antagonist	Pre-clinical	[89]
Liraglutide	GLP-1 receptor agonist	Pre-clinical	[90]
ObR antibody	Leptin receptor-blocker	Pre-clinical	[91]
Fenofibrate	PPARα agonist	Pre-clinical	[92]
Aleglitazar	PPARα/γ agonist	Pre-clinical	[93]
Lanifibranor	Pan-PPAR agonist	Pre-clinical	[94]
Enoxaparin	Anticoagulant	Pre-clinical	[95]
Telmisartan	PPAR-γ agonist and Ang II receptor antagonist	Pre-clinical	[96]
MAIT cell	Antimicrobial	Pre-clinical	[97]
Autologous macrophage therapy	Cell therapy for liver fibrogenic phenotype deactivation	Pre-clinical	[98]
Y27pPBHSA	ROCK inhibitor	Pre-clinical	[99]
Dabigatran	Thrombin inhibitor	Pre-clinical	[100]
ProAgio	Pro-apoptotic	Pre-clinical	[101, 102]
Apixaban	Anticoagulants	Pre-clinical	[103]
PTUPB	COX-2 and soluble epoxide hydrolase inhibitor	Pre-clinical	[104]
Teduglutide	GLP-2 receptor agonist	Pre-clinical	[105]

#### Antifibrotics

Structural changes and fibrogenesis contribute to progression of LC and PH. Although there is still not any approved antifibrotic drug, numerous efforts have been spent in developing candidate agents over the last decade. These efforts made it possible to clarify targets and pathways involved in liver fibrogenesis. Transforming growth factor β1 (TGF1) is a major contributor to HSCs activation, for which many different strategies have been proposed in the past. Monoclonal antibodies such as fresolimumab, currently in phase II clinical trial, can neutralize all isoforms of TFGβ [106]. Lysil-oxidase-like 2 (LOXL2) [107] plays a critical role in collagen cross-linking, rendering it more difficult to reabsorb. Simtuzumab is a monoclonal antibody against LOXL2 that, despite the initial preclinical results, showed no effect in phase II RCTs for primary sclerosing cholangitis, NASH fibrosis or cirrhosis, and HCV/HIV [50, 107, 108]. However, targeting LOX family members, still appears a promising strategy [109]. Galectin-3, from the lectin family, is a regulator of mRNA splicing and is associated with HSCs activation and hepatocellular carcinoma [110]. Finally, belapectin, an inhibitor of galectin-3, showed encouraging preclinical but controversial results in clinical trials [111, 112].

Indirect strategies for HSCs deactivation have raised considerable attention; antidiabetic drugs showed antifibrotic activity by reducing oxidative stress, inflammation, and consequently HSCs activation, such as antibodies against leptin receptor [91], metformin [113] and glucagon-like peptide-1 (GLP-1) receptor agonists (liraglutide, semaglutide) [48, 90, 114]. GLP-1a is used to treat type II diabetes, a co-morbidity frequently present in patients with NASH and that has been shown to be a suitable therapy for NASH resolution [115]. Additionally, the GLP-2 receptor, such as teduglutide, showed to be involved in the control of HSCs activation in a preclinical study based on mice treated with a high fat diet [105]. Therefore, GLP-1 and GLP-2 could be good candidate targets for therapy. Taurine is an essential amino acid that showed a reduction in HSCs contraction and activation [116]. A small randomized clinical study suggested intrahepatic activity with a reduction of HPGV by ~ 10% in 58% of CSPH patients [117].

Other drugs also have pleiotropic effects, including antifibrotic activity. Peroxisome proliferation-activated receptors (PPAR-α, β, γ/δ) are nuclear hormone receptors which regulates cholesterol synthesis, whose deregulation is involved in inflammation, insulin resistance, and fibrogenesis. Re-activation of these receptors showed encouraging results in reversing liver fibrosis, inflammation, steatosis, and extrahepatic complications of chronic liver disease [93, 118–123]. Among the many drugs targeting some of the PPAR, the pan-PPAR agonist lanifibranor, an indole sulfonamide derivative, has attracted much attention, since on top of our studies demonstrating marked effects in experimental cirrhosis [94]. Moreover, clinical studies suggest that lanifibranor may promote fibrosis regression in patients with NASH, although further studies are needed to confirm a therapeutic benefit in patients with advanced fibrosis [124]. PPARγ may reduce splanchnic angiogenesis and formation of portosystemic collaterals, as suggested by preclinical studies using aleglitazar, a dual PPAR α/γ agonist [93]. Telmisartan, a PPARγ agonist with antagonist properties on angiotensisn II type 1 receptor (AT1R), ameliorated PH by reducing inflammation, fibrosis, and vascular remodeling in bile-duct ligation and TAA cirrhotic rat models [96].

Recently, the cannabinoid receptors CB1 and CB2, which mediate glucose and lipid metabolism and insulin signaling, and are overexpressed in hepatic fibrosis [125], have been proposed as potential targets for the treatment of PH and NASH [126].

Structural and functional changes are intimately linked. Strategies against functional alterations such as reversing the changes in LSECs phenotype could contribute to deactivation of HSCs via crosstalk. For instance, soluble guanylyl cyclase (sGC) which is highly expressed in most liver cells, causes vasodilation via cyclic guanosine monophosphate (cGMP) production [127, 128]. sGC is physiologically activated by NO to cause vasodilation. Moreover, sGC can be targeted directly by drugs that act as stimulators or activators of sGC. Preclinical studies showed encouraging results preventing LSECs capillarization and reducing HSCs activation [129–131]. Riociguat, an sGMP stimulator that appears safe in advanced cirrhosis [132], has shown significant reductions in portal pressure, fibrosis, necroinflammation, and sinusoidal remodeling in bile duct-ligated rats [128], and it is close to enter phase II clinical trials. An alternative to Riociguat, is IW-1973, which showed similar properties together with reduced hepatic steatosis in a NASH model [133, 134]. Additionally, the sGC activator BAY 60–2770 showed promising results in a toxic-cirrhotic rat model [135]. Recently, it has been proposed that the protein ProAgio, by inducing the integrinα_v_β_3_-mediated HSCs apoptosis, may decrease liver fibrosis and reduce IHVR [101]. This type of approach acts on mechanosensing-dependent mechanisms that among others drive nuclear stretching and contribute to cell dysfunction as ECM stiffness increases with progressive liver fibrosis [102].

Thromboxane is a vasoconstrictor molecule which contributes to the increased vascular tone in advanced chronic liver disease (ACLD). Terutroban [78], an eicosanoid inhibitor that blocks thromboxane prostanoid receptor, reduced portal pressure by decreasing intrahepatic vascular resistance in experimental cirrhosis. However, ifetroban (a molecule with similar effects) was not found to modify these parameters in patients with cirrhosis treated for 3 months in a recent small RCT [136]. Finally, the dual cyclooxygenase-2 (COX2) soluble epoxide hydrolase inhibitor PTUPB was recently found to reduce liver fibrosis and PH in cirrhotic rats [104]. Previous experimental studies already shown that COX derived prostanoids modulate hepatic vascular tone in cirrhotic rats [137].

#### Anticoagulants

Occlusion of small hepatic veins as a result of endothelial injury is thought to cause parenchymal extinction contributing to tissue collapse and architectural distortion during the progression of cirrhosis [138]. This is in part the basis for proposing anticoagulants in patients with cirrhosis. Enoxaparin, a low molecular weight heparin like and rivaroxaban (a direct oral acting anticoagulant), decreases IHVR in preclinical studies in cirrhotic rat models with rats [95, 139]. In patients with cirrhosis on a waiting list for liver transplantation, 1-year treatment with enoxaparin prevented hepatic decompensation, decreased the episodes of portal vein thrombosis, decreased systemic inflammation and markers of bacterial translocation, and improved survival [140]. Finally, aspirin and other anti-platelet agents use was found to be associated with a decreased odds of hepatic fibrosis in patients at risk in a recent meta-analysis [141, 142].

### Agents Increasing the Bioavailability of NO in the Hepatic Circulation

#### Statins

Statins are drugs with pleiotropic effects on vascular biology that go beyond their lipid lowering capacity. Statins have been shown to act on several pathways, including Rho/ROCK and KLF2 [143], causing antifibrotic, anti-inflammatory, antioxidant, anti-proliferative, anti-coagulant, and vasculoprotective effects in experimental animals with several forms of LC and markedly increasing NO availability [144]. In clinical trials, simvastatin has been shown to decrease HVPG, improve liver function, and improve survival after variceal hemorrhage [145–147]. Moreover, the addition of statins (simvastatin/atorvastatin) to standard NSBB (propranolol) treatment improves the HVPG response [148–150]. Ongoing trials are currently assessing if some potential effects of statin delaying disease progression, decompensation, and death noticed in epidemiological studies [147] can be confirmed in randomized controlled studies [151, 152]. Despite concerns regarding risk of hepatic or muscular toxicity in ACLD patients, their safety profile has been shown to be acceptable even in decompensated Child–Pugh patients when administered at low doses (10–20 mg/day of simvastatin) [153]. Targeted delivery to LSEC, tested in preclinical models, may further enhance efficacy while decreasing the risk of adverse events [154].

#### Phosphodiesterase-5 (PDE5) Inhibitors

PDE5 is an enzyme which converts vasodilating cGMP into its inactive form and is increased in the cirrhotic liver. Its modulation using PDE5-inhibitors may have potential in the treatment of ACLD, as highlighted in a recent review [155]. In the clinical setting, results have been mixed, with some studies showing no change in HVPG [59, 156], while others observed a decrease [41, 60]. This class of drugs might be studied in early stage cirrhosis, but not in more advanced patients due to its effects decreasing systemic arterial pressure, which may be deleterious [59, 157].

Tetrahydrobiopterin (BH4) is a cofactor for NO synthesis which is downregulated in cirrhotic livers. However, a trial of a synthetic analog of BH4, sapropterin, did not demonstrate any beneficial hemodynamic or functional effect over 2 weeks of treatment [40].

#### Antioxidants

LSECs inflammation and injury are accompanied by increased oxidative stress which contributes to LSECs capillarization. Oxidative stress via ROS production in all liver cells decreases the bioavailability of NO [158] by scavenging it to form peroxynitrate, contributing to increase the hepatic vascular tone. Strategies based on antioxidants have tested intravenous ascorbic acid, recombinant human manganese superoxide dismutase (rMnSOD), and dark chocolate. SOD activity is reduced in cirrhosis [159], and a preclinical study showed that rMnSOD administration to cirrhotic rats decreased IHVR, portal pressure, and liver fibrosis. It further abolished endothelial dysfunction without affecting the systemic circulation [83]. Flavonoids are polyphenolic secondary metabolites of plants and are commonly assumed with the regular diet. Dark chocolate is reach in flavonoids, has a powerful antioxidant activity and increases NO bioavailability [160], and attenuates the increase in portal pressure associated with post-prandial hyperemia in patients with cirrhosis [46]. Other strategies targeting oxidative stress were based on the inhibition of NADPH, such as GKT137831 [161], angiotensin blocking agents (discussed in functional targets below). Resveratrol, a natural anti-oxidant with multiple effects on vascular biology, has also been found to decrease portal pressure, decrease fibrosis, and improve NO availability in rats with experimental cirrhosis [47, 162].

#### Renin–Angiotensin–Aldosterone System (RAAS)

Inhibitors include angiotensin-converting-enzyme inhibitors (ACEIs), angiotensin-receptor blockers (ARBs), and aldosterone antagonists (AAs). In advanced cirrhosis, after developing a hyperkinetic circulation, RAAS is activated in response to splanchnic and systemic vasodilation and contributes to sodium and water retention. Moreover, angiotensin II is a powerful vasoconstrictor that in HSCs has profibrotic effects [163]. Spironolactone is key in the management of ascites [38] and contributes to decrease the HVPG [164]. Trials of ACEIs or ARBs are heterogeneous regarding population, study drug, dosage, and treatment time and include small cohorts. A meta-analysis of RAAS modulators suggests a benefit of ARBs/ACEIs in early disease (Child–Pugh A) but raises concerns in more advanced patients due to hypotension-related complications [163]. Additional approaches for acting on RAAS include the MasR agonist and Janus-kinase-2 (JAK2) inhibitor. MasR agonist is a non-peptide agonist of angiotensin [1–7] able to reduce portal pressure by upregulation of NO and regulation of Rho-kinase [87]. JAK2 regulates RHOA/Roh-kinase activation from angiotensin II extrahepatically. Initial studies suggest that ruxolitinib, an inhibitor of JAK1 and JAK2, may cause a reduction of HVPG in cirrhosis [165].

#### Farnesoid X Receptors (FXR) Agonists

The FXR is a nuclear transcription factor responsive to bile acids. It is normally expressed in the liver but also in the intestine and kidney [166]. In preclinical models, steroidal (obeticholic acid (OCA)) or non-steroidal (cilofexor) FXR agonists decreased portal pressure, fibrosis, bacterial translocation and improved endothelial dysfunction via increased NO release [62, 63, 167–169]. In the clinical setting, obeticholic acid administered for 7 days to alcohol-induced chronic liver disease patients caused an HVPG reduction in some patients [170]; however, this agent may precipitate decompensation and death in advanced cirrhotic patients [171]. Recently, PX20606 reduced PH and showed anti-angiogenic effect [63].

#### Anti-angiogenics

In PH progression, angiogenesis is stimulated by several stimuli. Impaired microcirculation due to distorted intrahepatic circulation and sinusoidal pseudo-capillarization, and fibrosis stimulates expression of hypoxia-inducible factors (HIFs) and inflammation, with ensuing release of vascular endothelial grow factor (VEGF) and other angiogenic factors (placental growth factor (P1GF) and platelet-derived growth factor (PDGF)). The elevated pressure causes LSECs stretching and phenotypic dysregulation and activation of the Raf/MEK/ERK pathway [22, 172]. Among the anti-angiogenic compounds, sorafenib [173], sunitinib [174], regorafenib [175], and bivanib [176] reduced portal pressure, splanchnic neovascularization, and portosystemic shunting. Sorafenib, first multikinase inhibitor demonstrated to improve overall survival of HCC patients [177], has an anti-angiogenic activity by blocking the autophosphorylation of several receptors’ tyrosine kinases such as VEGFR1, 2, and 3; PDGFRβ; c-Kit; and RET and by inhibiting Raf kinase isoforms [178]. Despite being very effective in experimental cirrhosis, when used clinically, it has shown controversial results [179].

#### Rho-Kinase Inhibitors

Rho-kinase (ROCK) activity is correlated with activation of HSCs and endothelial dysfunction [31]. A clinical trial of fasudil, a ROCK inhibitor, has demonstrated an acute decrease in HVPG. However, it also caused systemic side-effects by significantly decreasing arterial pressure and systemic vascular resistance [180]. Future strategies may employ special delivery methods directly targeting HSCs [99].

#### Endothelin Antagonists

Endothelin is a potent vasoconstrictor molecule produced by the endothelium, which is increased in patients with cirrhosis. Moreover, in cirrhosis, there may be a change in the distribution and responsiveness of the different endothelin receptors [181]. This might explain the mixed results obtained so far with non-selective antagonists in clinical trials. While dual endothelin antagonists had beneficial effects on HRS and POPH [56], they did not significantly reduce HVPG [74, 182]. Specifically, blocking the ETA receptor might be a more promising strategy [43], and currently there is an ongoing open label trial of ambrisentan [42].

### Novel Extrahepatic Targets

There are promising strategies to modulate extrahepatic targets that may influence PH, either by means of decreasing the portal blood flow or by modulating extrahepatic mechanisms that finally influence liver disease (Table 2).

**Table 2:** Extrahepatic therapeutic compounds

Name	Mechanism	Exp Phase	Ref
Serelaxin	NO release	Phase II	[183]
Sorafenib	Tyrosine kinase inhibitor	Phase II	[179]
Albumin	Anti-inflammatory	Phase III	[184]
Metformin	Insulin sensitizer, anti-inflammatory	Phase IV	[185]
Fasudil	Rho-kinase inhibitor	Pre-clinical	[186]
Rapamycin	mTOR complex 1 inhibitor	Pre-clinical	[187]
t-TUCB	Soluble epoxide hydrolase inhibitor	Pre-clinical	[188]
Cerium oxide nanoparticles	Anti-inflammatory	Pre-clinical	[189]
Bifidobacterium pseudocatenulatum CECT7765	Anti-BT translocation	Pre-clinical	[190]
Probiotics (VSL#3)	Anti-BT translocation	Pre-clinical	[191]

#### Vasodilatatory and Antifibrotic

Relaxin is a vasodilatory and antifibrotic protein that is produced mainly by the ovary and breast in females and in the prostate in males. Important vasodilatory and antifibrotic properties have already been shown in pre-clinical studies [192–194], including a decrease in portal pressure. However, a small clinical trial with IV serelaxin, a human relaxin-2 analog, failed to show any decrease in HVPG after a 2-h infusion [183]. Innovative delivery methods may perhaps hold promise for serelaxin as a therapeutic agent [195].

#### Human Albumin

Serum albumin is produced by the liver and is markedly decreased in decompensated LC. Moreover, it is structurally and functionally abnormal, jeopardizing its antioxidant, scavenging, and immunomodulatory non-oncotic properties [196]. Consequently, albumin is considered a potential agent for the treatment of decompensated cirrhosis. A clinical trial of long-term treatment with weekly albumin infusion showed efficacy and improved survival [197]. However, there are concerns with the possibility of causing volume overload, especially in patients with NASH that frequently have associated cardiovascular disease. Such adverse effects were documented in a recent trial aimed at increasing albumin levels above 30 g/L in patients with decompensated cirrhosis [198]. A recent preclinical study in cirrhotic rats exploring the potential effect of the ROCK inhibitor coupled to a peptide-modified albumin carrier showed a reduced portal pressure and enhanced renal perfusion [99].

### Targeting Gut-Liver Axis

Bacterial translocation is a frequent event in decompensated cirrhosis with ascites. Translocation may cause endotoxemia and may be preceded by translocation of bacterial products, DAMPs and PAMPs, with associated pro-inflammatory responses that may enhance progression of the liver disease, cause systemic inflammation, and in the case of bacterial translocation result in sepsis or severe bacterial infections, such as spontaneous bacterial peritonitis.

The increased levels of endotoxin and pro-inflammatory cytokines contribute to endothelial dysfunction and vasoconstriction via ET-1 and vasoconstrictory prostanoids [199]. Limiting bacterial overgrowth and translocation, by means of non-absorbable antibiotics, NSBBs (that accelerate intestinal transit time and decrease intestinal permeability) and fecal transplantation may contribute to reduce liver inflammation and injury in LC [200]. Rifaximin [201] did not significantly decreased HVPG in patients with LC but decreased complications from PH [202]. Although rifaximin efficacy remains controversial [203], more studies are ongoing to clarify its application in LC [204]. Another approach to reduce bacterial overgrowth and translocation is administering probiotics. However, there is no evidence for now that probiotics have an impact on PH [205].

#### Cell Therapy: Parenchymal Extinction

Liver regeneration and fibrogenesis occur in parallel in chronic liver disease, although if liver injury continues, the process leads to the formation of cirrhosis, with regenerating liver nodules surrounded by fibrotic tracts, with a profoundly disturbed liver vascular architecture and IHVR. LSEC as well as quiescent HSCs and their crosstalk are important elements in support of the regenerative process. Cell therapy based on bone marrow transplantation was a promising approach to stimulate regeneration [206]. Bone marrow stem cells are mononucleated cells that include many different types of stem cells such as hematopoietic stem cells and mesenchymal stem cells. Hematopoietic cells can differentiate into hepatocytes, and a recent study showed improvements in liver function in patients with LC [207, 208]. Mesenchymal stem cells are easier to obtain and can exert different functions and present low levels of immunogenicity. A recent clinical trial in phase II showed that administration of mesenchymal stem cells improved liver fibrosis and Child–Pugh score in patients with alcoholic cirrhosis [209]. A novel approach used placental stem cells, with promising results in CCl4-cirrhotic rats [210].

A recent clinical trial studied the infusion of mature autologous monocyte-derived macrophages in patients with compensated cirrhosis with a change in MELD score at 90-day as primary outcome [98]. Cell therapy has also been shown to regulate microbial growth and function in the context of fibrosis [97]. This may be a promising opportunity for novel antifibrotic therapies.

## Conclusions

Knowledge of the PH mechanism underwent continuous development since the concept of splanchnic vasodilation contributing to increase portal pressure was introduced in the 1980s, providing for the first time a rationale for using splanchnic vasoconstrictors for the complications of PH. Since then, understanding the molecular mechanisms of disease progression and careful clinical observations have allowed identifying multiple targets to improve treatment and patients’ management. This has been paramount to ameliorate short and long-term prognosis of patients with cirrhosis, for which we now have disease-modifying therapies. While the current therapy and management are mainly focused on the prevention of the complications of PH and the decompensation of cirrhosis, the novel strategies are centered in reducing the IHVR and in preventing/reversing fibrosis.

Future therapy for PH is likely to involve multiple strategies aimed at acting on different mechanisms that act together and frequently synergistically promoting cirrhosis progression. New therapies that have shown promising results include the following: (i) statins, PPAR agonists, GLP-1 agonists, sGC activators and stimulators, enoxaparin, ribaroxaban, and dual or pan-FXR receptor agonists. Multiple RCTs are in progress, which makes the field of PH and cirrhosis a fascinating one, where rapid changes in management and prognosis are expected soon.

## Data Availability

Data sharing not applicable to this article as no datasets were generated or analysed during the current study.
